# Supplementary material for: Quality of reporting of cranial irradiation techniques in randomized controlled trials of primary brain tumors: A systematic review
Source: PLoS One. 2020 Nov 5;15(11):e0241566. doi: 10.1371/journal.pone.0241566 (PMC7644083; doi:10.1371/journal.pone.0241566)
Supplement: S1 Table — (DOCX) [file pone.0241566.s001.docx]

S1 Table. Search strategy

| Database | Search term |
| --- | --- |
| MEDLINE (via PubMed) | (Brain neoplasms[Mesh] OR brain neoplas*[tiab] OR brain cancer*[tiab] OR brain tumour*[tiab] OR intracranial neoplas*[tiab] OR intracranial cancer*[tiab] OR intracranial tumour*[tiab]) AND (“radiotherapy”[Mesh] OR radiotherapy[tiab] OR radiotherapy*[tiab] OR radiation[tiab] OR irradiation[tiab] OR RT[tiab])  AND ("1999/01/01"[PDat] : "2019/11/30"[PDat]) AND (randomized controlled trial [ptyp]) |
| EMBASE | (‘Brain neoplasms’/exp OR ‘brain neoplas*’:ab,ti OR ‘brain cancer*’:ab,ti OR ‘brain tumour*’:ab,ti OR ‘intracranial neoplas*’:ab,ti OR ‘intracranial cancer*’:ab,ti OR ‘intracranial tumour*’:ab,ti) AND (‘radiotherapy’/exp OR radiotherapy:ab,ti OR radiotherapy*:ab,ti OR radiation:ab,ti OR irradiation:ab,ti OR RT:ab,ti) AND [randomized controlled trial]/lim AND [1999-2019]/py AND [article]/lim |
