# Supplementary material for: Quality of reporting of cranial irradiation techniques in randomized controlled trials of primary brain tumors: A systematic review
Source: PLoS One. 2020 Nov 5;15(11):e0241566. doi: 10.1371/journal.pone.0241566 (PMC7644083; doi:10.1371/journal.pone.0241566)
Supplement: S2 Table — (DOCX) [file pone.0241566.s002.docx]

S2 Table. List of included studies

| Study ID | First Author | Year of Publication | Journal | Title |
| --- | --- | --- | --- | --- |
| 1 | Zhang | 2015 | Zhonghua Yi Xue Za Zhi | Randomized controlled study of limited margins IMRT and temozolomide chemotherapy in patients with malignant glioma |
| 2 | Roa | 2015 | J Clin Oncol | International Atomic Energy Agency randomized phase III study of radiation therapy in elderly and/or frail patients with newly diagnosed glioblastoma multiforme |
| 3 | Lannering | 2012 | J Clin Oncol | Hyperfractionated versus conventional radiotherapy followed by chemotherapy in standard risk medulloblastoma: results from the randomized multicenter HIT-SIOP PNET 4 trial |
| 4 | Chinot | 2014 | N Engl J Med | Bevacizumab plus radiotherapy-temozolomide for newly diagnosed glioblastome |
| 5 | Blumenthal | 2015 | Int J Clin Oncol | A phase III study of radiation therapy (RT) and O^6^-benzylguanine + BCNU versus RT and BCNU alone and methylation status in newly diagnosed glioblastoma and gliosarcoma: Southwest Oncology Group (SWOG) study S0001. |
| 6 | Zaghloul | 2014 | Radiother Oncol | Hypofractionated conformal radiotherapy for pediatric diffuse intrinsic pontine glioma (DIPG): a randomized controlled trial |
| 7 | Hofland | 2014 | Acta Oncol | Neoadjuvant bevacizumab and irinotecan versus bevacizumab and temozolomide followed by concomitant chemoradiotherapy in newly diagnosed glioblastoma multiforme. A randomized phase II study |
| 8 | Shaw | 2012 | J Clin Oncol | Randomized trial of radiation therapy plus procarbazine, lomustine and vincristine chemotherapy for supratentorial adult low grade glioma: initial results of RTOG 9802 |
| 9 | Tarbell | 2013 | J Clin Oncol | High-risk medulloblastoma: a pediatric oncology group randomized trial of chemotherapy before or after radiation therapy (POG 9031) |
| 10 | Solomon | 2013 | BMC Cancer | Radiotherapy plus nimotuzumab or placebo in the treatment of high grade glioma patients: results from a randomized double trial |
| 11 | Shibui | 2013 | Cancer Chemother Pharmacol | Randomized trial of chemoradiotherapy and adjuvant chemotherapy with nimustine (ACNU) versus nimustine plus procarbazine for newly diagnosed anaplastic astrocytoma and glioblastoma (JCOG0305) |
| 12 | Cairncross | 2006 | J Clin Oncol | Phase III trial of chemotherapy plus radiotherapy compared with radiotherapy alone for pure and mixed anaplastic oligodendroglioma: Intergroup Radiation Therapy Oncology Group Trial 9402 |
| 13 | Malmstrom | 2012 | Lancet Oncol | Temozolomide versus standard 6 week radiotherapy versus hypofractionated radiotherapy in patients older than 60 years with glioblastoma: the Nordic randomized phase 3 trial |
| 14 | Baumert | 2016 | Lancet Oncol | Temozolomide chemotherapy versus radiotherapy in high risk low grade glioma (EORTC 22033-26033): a randomized open label phase 3 intergroup study |
| 15 | Nabors | 2012 | Cancer | A safety run in and randomized phase 2 study of cilengitide combined with chemoradiation for newly diagnosed glioblastoma (NABTT 0306) |
| 16 | Zhai | 2012 | Nan Fang Yi Ke Da Xue Xue Bao | Comparison of two regimens of postoperative concurrent chemoradiotherapy in adult patients with grade III-IV cerebral gliomas |
| 17 | Wick | 2009 | J Clin Oncol | NOA-04 randomized phase III trial of sequential radiochemotherapy of anaplastic glioma with procarbazine, lomustine and vincristine or temozolomide |
| 18 | Grabenbauer | 2009 | Int J Radiat Oncol Biol Phys | Effects of concurrent topotecan and radiation on 6 month progression free survival in the primary treatment of glioblastoma multiforme |
| 19 | Clarke | 2009 | J Clin Oncol | Randomized phase II trial of chemoradiotherapy followed by either dose dense or metronomic temozolomide for newly diagnosed glioblastoma |
| 20 | Kocher | 2008 | Strahlenther Onkol | Randomized study of postoperative radiotherapy and simultaneous temozolomide without adjuvant chemotherapy for glioblastoma |
| 21 | Baumert | 2008 | Radiother Oncol | EORTC 22972026881/MRI BR 10 trial: fractionated stereotactic boost following conventional radiotherapy of high grade gliomas. Clinical and quality assurance results of the stereotactic boost arm |
| 22 | Packer | 2006 | J Clin Oncol | Phase III study of craniospinal radiation therapy followed by adjuvant chemotherapy for newly diagnosed average risk medulloblastoma |
| 23 | van den Bent | 2006 | J Clin Oncol | Adjuvant procarbazine, lomustine, and vincristine improves progression-free survival but not overall survival in newly diagnosed anaplastic oligodendrogliomas and oligoastrocytomas: a randomized European Organisation for Research and Treatment of Cancer phase III trial |
| 24 | Macdonald | 2005 | Cancer | Phase II study of high dose chemotherapy before radiation in children with newly diagnosed high grade astrocytoma: final analysis of Children’s Cancer Group Study 9933 |
| 25 | Marshall | 2006 | J Neurooncol | Ototoxicity of cisplatin plus standard radiation therapy vs accelerated radiation therapy in glioblastoma patients |
| 26 | Stupp | 2005 | N Engl J Med | Radiotherapy plus concomitant and adjuvant temozolomide for glioblastoma |
| 27 | Souhami | 2004 | Int J Radiat Oncol Biol Phys | Randomized comparison of stereotactic radiosurgery followed by conventional radiotherapy with carmustine to conventional radiotherapy with carmustine for patients with glioblastoma multiforme: report of Radiation Therapy Oncology Group 93-05 protocol |
| 28 | Roa | 2004 | J Clin Oncol | Abbreviated course of radiation therapy in older patients with glioblastoma multiforme: a prospective randomized clinical trial |
| 29 | Phillips | 2003 | Radiother Oncol | A randomized trial comparing 35Gy in ten fractions with 60Gy in 30 fractions of cerebral irradiation for glioblastoma multiforme and older patients with anaplastic astrocytoma |
| 30 | Taylor | 2003 | J Clin Oncol | Results of a randomized study of preradiation chemotherapy versus radiotherapy alone for nonmetastatic medulloblastoma: The International Society of Paediatric Oncology / United Kingdom Children’s Cancer Study Group PNET-3 Study |
| 31 | Grossman | 2003 | J Clin Oncol | Phase III study comparing three cycles of infusional carmustine and cisplatin followed by radiation therapy with radiation therapy and concurrent carmustine in patients with newly diagnosed supratentorial glioblastoma multiforme: Eastern Cooperative Oncology Group Trial 2394 |
| 32 | Selker | 2002 | Neurosurgery | The Brain Tumor Cooperative Group NIH Trial 87-01: a randomized comparison of surgery, external radiotherapy, carmustine versus surgery, interstitial radiotherapy boost, external radiation therapy, and carmustine |
| 33 | Shaw | 2002 | J Clin Oncol | Prospective randomized trial of low versus high dose radiation therapy in adults with supratentorial low grade glioma: initial report of a North Central Cancer Treatment Group / Radiation Therapy Oncology Group / Eastern Cooperative Oncology Group study |
| 34 | Prados | 2001 | Int J Radiat Oncol Biol Phys | Phase III trial of accelerated hyperfractionation with or without difluromethylornithine (DFMO) versus standard fractionated radiotherapy with or without DFMO for newly diagnosed patients with glioblastoma multiforme |
| 35 | Kortmann | 2000 | Int J Radiat Oncol Biol Phys | Postoperative neoadjuvant chemotherapy before radiotherapy as compared to immediate radiotherapy followed by maintenance chemotherapy in the treatment of medulloblastoma in childhood: results of the German prospective randomized trial HIT’91 |
| 36 | Mandell | 1999 | Int Radiat Oncol Biol Phys | There is no role for hyperfractionated radiotherapy in the management of children with newly diagnosed diffuse intrinsic brainstem tumors: results of a pediatric oncology group phase III trial comparing conventional vs hyperfractionated radiotherapy |
| 37 | Karim | 2002 | Int J Radiat Oncol Biol Phys | Randomized trial on the efficacy of radiotherapy for cerebral low grade glioma in the adult: European Organization for Research and Treatment of Cancer Study 22845 with the Medical Research Council study BR04: an interim analysis |
| 38 | Gilbert | 2014 | N Engl J Med | A randomized trial of bevacizumab for newly diagnosed glioblastoma |
| 39 | Herrlinger | 2016 | J Clin Oncol | Bevacizumab plus irinotecan versus temozolomide in newly diagnosed O6-Methylguanine-DNA Methyltransferase Nonmethylated Glioblastoma: The randomized GLARIUS trial |
| 40 | Chauffert | 2014 | Ann Oncol | Randomized phase II trial of irinotecan and bevacizumab as neo-adjuvant and adjuvant to temozolomide based chemoradiation compared with temozolomide chemoradiation for unresectable glioblastoma: final results of the TEMAVIR study from ANOCEF |
| 41 | Elinzano | 2018 | Am J Clin Oncol | PPX and concurrent radiation for newly diagnosed glioblastoma without MGMT methylation: a randomized phase II study: BrUOG 244 |
| 42 | Balana | 2016 | J Neurooncol | Bevacizumab and temozolomide versus temozolomide alone as neoadjuvant treatment in unresected glioblastoma: the GENOM 009 randomized phase II trial |
| 43 | Lee | 2015 | Clin Cancer Res | A multicenter phase II randomized noncomparative clinical trial of radiation and temozolomide with or without vandetanib in newly diagnosed glioblastoma patients |
| 44 | Armstrong | 2013 | J Clin Oncol | Net clinical benefit analysis of radiation therapy oncology group 0525: a phase III trial comparing conventional adjuvant temozolomide with dose-intensive temozolomide in patients with newly diagnosed glioblastoma |
| 45 | Nabors | 2015 | Neuro Oncol | Two cilengitide regimens in combination with standard treatment for patients with newly diagnosed glioblastoma and unmethylated MGMT gene promoter: results of the open label controlled randomized phase II CORE study |
| 46 | Stupp | 2014 | Lancet Oncol | Cilengitide combined with standard treatment for patients with newly diagnosed glioblastoma with methylated MGMT promoter (CENTRIC EORTC 26071-22072 study): a multicenter, randomized open-label phase 3 trial |
| 47 | Medical research council brain tumor working party | 2001 | J Clin Oncol | Randomized trial of procarbazine, lomustine and vincristine in the adjuvant treatment of high grade astrocytoma: a Medical Research Council trial |
| 48 | Zhou | 2015 | Asian Pac J Cancer Prev | Clinical observation of three dimensional conformal radiotherapy with tamoxifen in treatment of postoperative malignant glioma |
| 49 | Wygoda | 2006 | Hybridoma | Use of monoclonal anti-EGFR antibody in the radioimmunotherapy of malignant gliomas in the context of EGFR expression in grade III and IV tumors |
| 50 | Wick | 2012 | Lancet Oncol | Temozolomide chemotherapy alone versus radiotherapy alone for malignant astrocytoma in the elderly: the NOA-08 randomized phase 3 trial |
| 51 | Wick | 2016 | Clin Cancer Res | Phase II study of radiotherapy and temsirolimus versus radiochemotherapy with temozolomide in patients with newly diagnosed glioblastoma without MGMT promoter hypermethylation (EORTC 26082) |
| 52 | Weller | 2003 | J Clin Oncol | Neuro-Oncology working group 01 trial of nimustine plus teniposide versus nimustine plus cytarabine chemotherapy in addition to involved field radiotherapy in the first line treatment of malignant glioma |
| 53 | Wakabayashi | 2018 | J Neurooncol | JCOG0911 INTEGRA study: a randomized screening phase II trial of interferon β plus temozolomide in comparison with temozolomide alone for newly diagnosed glioblastoma |
| 54 | Thomas | 2000 | J Clin Oncol | Low-stage medulloblastoma: final analysis of trial comparing standard-dose with reduced dose neuraxis irradiation |
| 55 | Szczepanek | 2013 | Neurol Neurochir Pol | Efficacy of concomitant and adjuvant temozolomide in glioblastoma treatment: a multicenter randomized study |
| 56 | Sotelo | 2006 | Ann Intern Med | Adding chloroquine to conventional treatment for glioblastoma multiforme: a randomized double blind placebo controlled trial |
| 57 | Shim | 2013 | Childs Nerv Syst | Treatment strategy for intracranial primary pure germinoma |
| 58 | Sharma | 2003 | Neurol India | Local control of high grade gliomas with limited volume irradiation versus whole brain irradiation |
| 59 | Prados | 1999 | Int J Radiat Oncol Biol Phys | A phase 3 randomized study of radiotherapy plus procarbazine, CCNU, and vincristine (PCV) with or without BUdR for the treatment of anaplastic astrocytoma: a preliminary report of RTOG 9404 |
| 60 | Perry | 2017 | N Engl J Med | Short course radiation plus temozolomide in elderly patients with glioblastoma |
| 61 | Montemor | 2008 | Onkologie | Concurrent chemoradiotherapy with weekly paclitaxel in malignant cerebral glioma treatment |
| 62 | Menei | 2005 | Neurosurgery | Local and sustained delivery of 5-fluorouracil from biodegradable microspheres for the radiosensitization of malignant glioma: a randomized phase II trial |
| 63 | Mao | 2015 | Chin Med J | Does early postsurgical temozolomide plus concomitant radiochemotherapy regimen have any benefit in newly diagnosed glioblastoma patients? A multicenter, randomized parallel open-label phase II clinical trial |
| 64 | Malmstrom | 2017 | Acta Oncol | Postoperative neoadjuvant temozolomide before radiotherapy versus standard radiotherapy in patients 60 years or younger with anaplastic astrocytoma or glioblastoma: a randomized trial |
| 65 | Mallick | 2018 | J Neurooncol | Hypofractionated accelerated radiotherapy (HART) with concurrent and adjuvant temozolomide in newly diagnosed glioblastoma: a phase II randomized trial (HART-GBM trial) |
| 66 | Ladenstein | 2017 | Lancet Oncol | Busulfan and melphalan versus carboplatin, etoposide, and melphalan as high dose chemotherapy for high risk neuroblastoma (HR-NBL1/SIOPEN): an international randomized multi-arm open-label phase 3 trial |
| 67 | Kong | 2017 | Oncotarget | Phase III randomized trial of autologous cytokine induced killer cell immunotherapy for newly diagnosed glioblastoma in Korea |
| 68 | Kochii | 2000 | J Neurooncol | Randomized comparison of intra-arterial versus intravenous infusion on ACNU for newly diagnosed patients with glioblastoma |
| 69 | Kim | 2011 | J Neurooncol | Radiotherapy followed by adjuvant temozolomide with or without neoadjuvant ACNU-CDDP chemotherapy in newly diagnosed glioblastomas: a prospective randomized controlled multicenter phase III trial |
| 70 | Keime-Guibert | 2007 | N Engl J Med | Radiotherapy for glioblastoma in the elderly |
| 71 | Jennings | 2002 | J Clin Oncol | Preradiation chemotherapy in primary high risk brainstem tumors: phase II study CCG-9941 of the Children’s Cancer Group |
| 72 | Jalali | 2017 | JAMA Oncol | Efficacy of stereotactic conformal radiotherapy vs conventional radiotherapy on benign and low grade brain tumors: a randomized clinical trial |
| 73 | Immonen | 2004 | Mol Ther | AdvHSV-tk gene therapy with intravenous ganciclovir improves survival in human malignant glioma: a randomized controlled study |
| 74 | Hildebrand | 2008 | Eur J Cancer | Adjuvant dibromodulcitol and BCNU chemotherapy in anaplastic astrocytoma: results of a randomized European Organisation for Research and Treatment of Cancer phase III study (EORTC study 26882) |
| 75 | Herrlinger | 2019 | Lancet | Lomustine-temozolomide combination therapy versus standard temozolomide therapy in patients with newly diagnosed glioblastoma with methylated MGMT promoter (CeTeG/NOA-09): a randomized open-label phase 3 trial |
| 76 | Henriksson | 2006 | J Neurooncol | High-grade astrocytoma treated concomitantly with estramustine and radiotherapy |
| 77 | Chinnaiyan | 2018 | Neuro Oncol | A randomized phase II study of everolimus in combination with chemoradiation in newly diagnosed glioblastoma: results of NRG Oncology RTOG 0913 |
| 78 | Chang | 2017 | Neuro Oncol | Phase III randomized study of radiation and temozolomide versus radiation and nitrosourea therapy for anaplastic astrocytoma: results of NRG Oncology RTOG 9813 |
| 79 | Buckner | 2001 | Cancer | A phase III study of radiation therapy plus carmustine with or without recombinant interferon alpha in the treatment of patients with newly diagnosed high grade glioma |
| 80 | Buckner | 2006 | J Clin Oncol | Phase III trial of carmustine and cisplatin compared with carmustine alone and standard radiation therapy or accelerated radiation therapy in patients with glioblastoma multiforme: North central cancer treatment group 93-72-52 and Southwest oncology group 9503 trials |
| 81 | Bertolone | 2003 | J Neurooncol | Combined modality therapy for poorly differentiated gliomas of the posterior fossa in children: a Children’s Cancer Group report |
| 82 | Athanassiou | 2005 | J Clin Oncol | Randomized phase II study of temozolomide and radiotherapy compared with radiotherapy alone in newly diagnosed glioblastoma multiforme |
| 83 | Ali | 2018 | J Neurooncol | NRG oncology RTOG 9006: a phase III randomized trial of hyperfractionated radiotherapy (RT) and BCNU versus standard RT and BCNU for malignant glioma patients |
| 84 | Abd El-Aal | 2005 | J Egypt Natl Canc Inst | Medulloblastoma: conventional radiation therapy in comparison to chemoradiation therapy in the post-operative treatment of high risk patients |
| 85 | van den Bent | 2017 | Lancet | Interim results from the CATNON trial (EORTC study 26053-22054) of treatment with concurrent and adjuvant temozolomide for 1p/19q non-co-deleted anaplastic glioma: a phase 3 randomized open label intergroup study |
