# Supplementary material for: Quality of reporting of cranial irradiation techniques in randomized controlled trials of primary brain tumors: A systematic review
Source: PLoS One. 2020 Nov 5;15(11):e0241566. doi: 10.1371/journal.pone.0241566 (PMC7644083; doi:10.1371/journal.pone.0241566)
Supplement: S3 Table — (DOCX) [file pone.0241566.s003.docx]

S3 Table. Characteristics of included studies

| Study ID | Number of patients included in study (N) | Histology (according to trial report) | Female Gender (%) | Median age*  (years) | Number of study arms | Study arms | Median follow up (months) | Primary outcome | Meet pre-specified endpoint |
| --- | --- | --- | --- | --- | --- | --- | --- | --- | --- |
|  |  |  |  |  |  |  |  |  |  |
| 1 | 50 | High grade gliomas | 38 | 59 | 2 | Standard GTV to CTV margins expansion vs Limited GTV to CTV margins expansion | NR | PFS | Yes |
| 2 | 98 | GBM | 54 | NR | 2 | 40Gy/15 fractions of RT vs 25Gy/5 fractions of RT | 6 | OS | Yes |
| 3 | 340 | Medulloblastoma | 38 | NR | 2 | Postoperative standard fractionated RT followed by chemotherapy vs Postoperative hyperfractionated RT followed by chemotherapy | 58 | EFS | No |
| 4 | 921 | GBM | 37 | 57 | 2 | Placebo + RT and temozolomide vs Bevacizumab + RT and temozolomide | 14 | OS | No |
| 5 | 183 | GBM | 39 | 56 | 2 | RT + BCNU vs RT + O6 Benzylguanine + BCNU | NR | OS | No |
| 6 | 71 | DIPG | 34 | 8 | 2 | Conventional RT vs Hypofractionated RT | 9 | OS | No |
| 7 | 63 | GBM | 38 | 60 | 2 | Bevacizumab + temozolomide + chemoradiotherapy vs Bevacizumab + irinotecan + chemoradiotherapy | 31 | PFS | No |
| 8 | 251 | Low grade glioma | NR | 41 | 2 | RT alone vs RT + PCV | 49 | OS | No |
| 9 | 112 | Medulloblastoma | 41 | NR | 2 | RT followed by chemotherapy vs Chemotherapy followed by RT | 77 | EFS | No |
| 10 | 70 | AA, GBM | 43 | 45 | 2 | RT + placebo vs RT + nimotuzumab | NR | OS | Yes |
| 11 | 111 | AA, GBM | 41 | 55 | 2 | RT + ACNU vs RT + PCZ + ACNU | 20 | OS | No |
| 12 | 289 | AO | 57 | 43 | 2 | RT vs RT + PCV | 61 | OS | No |
| 13 | 291 | GBM | 41 | 70 | 3 | Standard fractionated RT vs TMZ vs Hypofractionated RT | NR | OS | Yes |
| 14 | 707 | Low grade gliomas | 29 | 45 | 2 | RT vs TMZ | 48 | PFS | No |
| 15 | 112 | GBM | 41 | 55 | 2 | RT + chemotherapy vs RT + TMZ + EMD | NR | OS | Yes |
| 16 | 96 | High grade gliomas | 44 | NR | 2 | Teniposide + semustine + RT vs TMZ + RT | NR | OS | Yes |
| 17 | 318 | AA, AO | 50 | 43 | 2 | Chemo 🡪 RT vs RT 🡪 chemo | NR | TTF | No |
| 18 | 52 | GBM | 24 | 52 |  | RT vs Topotecan + RT | 14 | PFS | No |
| 19 | 85 | GBM | 34 | 56 | 2 | Chemoradiotherapy 🡪 Dose Dense TMZ vs chemoradiotherapy 🡪 Metronomic TMZ | 19 | OS | Yes |
| 20 | 65 | GBM | 32 | 59 | 2 | RT vs TMZ + RT | NR | PFS | No |
| 21 | 25 | High grade gliomas | NR | NR | 2 | Conventional RT vs Conventional RT + Fractionated stereotactic boost | 39 | OS | No |
| 22 | 421 | Medulloblastoma | 41 | NR | 2 | Craniospinal RT + cyclophosphamide, cisplatin and vincristine vs Craniospinal RT + lomustine, cisplatin and vincristine | 60 | TTF | No |
| 23 | 368 | AA, AO | 42 | 49 | 2 | RT alone vs RT + PCV | 60 | OS | No |
| 24 | 76 | High grade gliomas | 43 | 12 | 3 | Carboplatin/etoposide + RT 🡪 maintenance chemotherapy  Ifosfamide/etoposide + RT 🡪 maintenance chemotherapy  Cyclophosphamide/etoposide + RT 🡪 maintenance chemotherapy | NR | Tumor response | No |
| 25 | 230 | GBM | 37 | 54 | 2 | No cisplatin-based Chemotherapy + standard RT vs Cisplatin-based Chemotherapy + RT | NR | Ototoxicity | Yes |
| 26 | 573 | GBM | 37 | 57 | 2 | RT alone vs TMZ + RT | 28 | OS | Yes |
| 27 | 203 | GBM | 37 | 56 | 2 | RT alone vs SRS + RT | 61 | OS | No |
| 28 | 100 | GBM | 50 | 72 | 2 | 6 week RT vs 3 week RT | NR | OS | Yes |
| 29 | 68 | AA, GBM | 28 | 59 | 2 | 60Gy/30 fractions RT vs 35Gy/10 fractions RT | NR | OS | No |
| 30 | 179 | Medulloblastoma | 38 | 8 | 2 | RT alone vs Chemotherapy + RT | 65 | OS | No |
| 31 | 219 | GBM | 45 | 55 | 2 | BCNU + RT vs BCNU + cisplatin 🡪 RT | 40 | OS | No |
| 32 | 270 | High grade glioma | 40 | NR | 2 | RT, BCNU vs RT, interstitial RT boost, BCNU | NR | OS | No |
| 33 | 203 | Low grade gliomas | 43 | 40 | 2 | 50.4Gy/28 fractions RT vs 64.8Gy/36 fractions RT | 77 | OS | No |
| 34 | 231 | GBM | 41 | 57 | 4 | 59.4Gy/33 fractions vs Accelerated hyperfractionation (70.4Gy/44 fractions) vs Accelerated hyperfractionation (70.4Gy/44 fractions) + DFMO (difluromethylornithine) vs 59.4Gy/33 fractions + DFMO | NR | OS  PFS | No |
| 35 | 137 | Medulloblastoma | 34 | 7 | 2 | RT + concomitant vincristine 🡪 maintenance chemotherapy vs Neoadjuvant chemotherapy 🡪 RT | 30 | PFS | No |
| 36 | 130 | DIPG | 55 | 6 | 2 | 54Gy/30 fractions vs 70.2Gy/60 fractions twice-daily | NR | OS  PFS | No |
| 37 | 311 | Low grade glioma | 38 | NR | 2 | No postoperative RT vs Early RT within 8 weeks of the day of surgery | 60 | OS  TTP | OS – no  TTP – yes |
| 38 | 637 | GBM | 40 | NR | 2 | RT + TMZ vs RT+ TMZ, with Bevacizumab | 21 | OS PFS | No |
| 39 | 182 | GBM | 33 | 56 | 2 | RT + TMZ 🡪 TMZ vs Bevacizumab + RT 🡪 maintenance bevacizumab + irinotecan | NR | PFS at 6 months | Yes |
| 40 | 60 | GBM | 41 | 61 | 2 | RT + TMZ 🡪TMZ vs Neo-adjuvant intravenous Bevacizumab and irinotecan 🡪 RT + TMZ and bevacizumab 🡪 adjuvant Bevacizumab and irinotecan x 6 months. | NR | PFS at 6 months | No |
| 41 | 551 | GBM | 38 | 62 | 2 | TMZ + RT vs PPX + RT | 12 | PFS | No |
| 42 | 102 | GBM | 40 | 62 | 2 | Neoadjuvant TMZ + bevacizumab 🡪 RT + TMZ + bevacizumab and adjuvant TM vs Neoadjuvant TMZ 🡪 RT + TMZ and adjuvant TMZ | NR | Tumour response | No |
| 43 | 106 | GBM | 60 | 57 | 2 | Concurrent TMZ and RT 🡪adjuvant TMZ vs Vandetanib + concurrent TMZ and RT 🡪 adjuvant TMZ | NR | OS | No |
| 44 | 182 | GBM | 48 | 58 | 2 | Concurrent TMZ and RT 🡪 standard dose adjuvant TMZ vs Concurrent TMZ and RT 🡪 dose-intensive adjuvant TMZ | NR | Neurocognitive function | Yes |
| 45 | 265 | GBM | 47 | 57 | 3 | TMZ + RT followed by adjuvant RT vs EMD (standard dose) with TMZ + RT vs EMD (intensive dose), with TMZ + RT | NR | OS | No |
| 46 | 545 | GBM | 53 | 58 | 2 | TMZ + RT 🡪 TMZ alone vs TMZ + RT 🡪 TMZ with EMD | NR | OS | No |
| 47 | 674 | AA | 33 | NR | 2 | RT vs RT-PCV | 36 | OS | No |
| 48 | 60 | AA, GBM | 43 | 46 | 2 | RT + TMZ vs RT + Tamoxifen | NR | OS | No |
| 49 | 18 | High grade gliomas | NR | NR | 2 | Teleradiotherapy alone vs Postoperative teleradiotherapy with radioimmunotherapy | NR | OS  DFS | No |
| 50 | 373 | AA, GBM | 48 | 72 | 2 | RT vs TMZ | 25 | OS | Yes |
| 51 | 111 | GBM | 36 | 56 | 2 | RT + TMZ 🡪 adjuvant TMZ vs RT + weekly temsirolimus | 33 | OS at 12 months | No |
| 52 | 375 | High grade gliomas | 37 | 51 | 2 | ANCU + Ara-C + RT vs ANCU + VM26 + RT | 35 | OS | No |
| 53 | 122 | GBM | 25 | 61 | 2 | TMZ + RT vs TMZ vs TMZ + IFNβ + RT vs maintenance TMZ+IFNβ | 24 | OS | No |
| 54 | 126 | Medulloblastoma | NR | NR | 2 | Standard-dose (36Gy/20fractions) vs Reduced-dose craniospinal RT (23.4Gy/13fractions); Posterior fossa received 54Gy in both arms | NR | Isolated neuraxis recurrence | No |
| 55 | 58 | GBM | 42 | 55 | 2 | RT alone vs TMZ 🡪 RT +TMZ 🡪 TMZ | 33 | OS | Yes |
| 56 | 30 | GBM | 43 | 43 | 2 | BCNU + RT vs BCNU + RT, with chloroquine | NR | Survival after surgery | No |
| 57 | 28 | Intracranial primary pure germi-  nomas | 32 | 16 | 2 | RT vs Chemotherapy 🡪 RT | 60 | OS | No |
| 58 | 50 | High grade gliomas | NR | NR | 2 | Whole brain RT vs Limited-field RT | NR | Local control  OS | No |
| 59 | 281 | AA | 41 | NR | 2 | RT + PCV vs RT/BUdR + PCV | 36 | OS  TTP | No |
| 60 | 562 | GBM | 39 | 73 | 2 | RT vs RT + TMZ 🡪 TMZ | 17 | OS | Yes |
| 61 | 61 | AA, GBM | NR | 46 | 2 | Adjuvant RT vs adjuvant RT+ weekly paclitaxel | NR | OS | No |
| 62 | 95 | AA, GBM | 34 | 56 | 2 | 5-Fluorouracil releasing microspheres + RT vs RT | NR | OS | No |
| 63 | 99 | GBM | 33 | 51 | 2 | Concurrent chemoradiotherapy + early post-surgical TMZ vs Concurrent chemoradiotherapy | NR | OS | Yes |
| 64 | 144 | AA, GBM | 38 | 53 | 2 | TMZ 🡪 RT vs RT | 20 | OS | No |
| 65 | 89 | GBM | 37 | 45 | 2 | Conventional fractionated RT vs Hypofractionated accelerated RT | 11 | OS | No |
| 66 | 676 | Neuroblastoma | 38 | 3 | 2 | Busulphan/melphalan + RT vs Carboplatin/etoposide/melphalan + RT | 86 | EFS | Yes |
| 67 | 180 | GBM | 43 | 55 | 2 | Cytokine induced killer cells/TMZ chemoradiotherapy vs TMZ/chemoradiotherapy | NR | PFS | Yes |
| 68 | 82 | GBM | 40 | 57 | 2 | Intraarterial ACNU + RT vs intravenous ACNU + RT | NR | OS  PFS | No |
| 69 | 92 | GBM | 68 | 51 | 2 | ACNU + CCDP 🡪 RT 🡪 TMZ vs RT 🡪 TMZ | NR | OS | No |
| 70 | 85 | AA, GBM | 37 | 74 | 2 | Supportive care vs RT | 5 | OS | Yes |
| 71 | 63 | High grade gliomas | 86 | 6 | 2 | Carboplatin/etoposide/vincristine + RT vs cisplatin, cyclophosphamide, etoposide, vincristine + RT | NR | EFS | No |
| 72 | 200 | Low grade gliomas | 34 | 13 | 2 | Stereotactic conformal RT vs Conventional RT | 61 | Change in neuropsycological/neuroendocrine function | Yes |
| 73 | 36 | High grade gliomas | 33 | 55 | 2 | ADvHSV-tk gene therapy/GCV + RT vs RT | NR | OS | Yes |
| 74 | 193 | AA | 52 | 42 | 2 | RT vs RT+DBD/BCNU | NR | OS | No |
| 75 | 141 | GBM | 40 | 58 | 2 | Lomustine/TMZ + RT vs TMZ +RT | NR | OS | Yes |
| 76 | 122 | AA | 37 | 55 | 2 | Estramustine + RT vs RT | 62 | OS | No |
| 77 | 171 | GBM | 40 | NR | 2 | Chemoradiotherapy vs chemoradiotherapy + everolimus | 28 | PFS | No |
| 78 | 196 | AA | NR | 43 | 2 | TMZ + RT vs NU + RT  NU = nitrosurea | 43 | TTP | No |
| 79 | 275 | AA, GBM | 41 | 57 | 2 | BCNU + RT vs BCNU/IFN-alpha + RT | NR | OS | No |
| 80 | 451 | GBM | 37 | NR | 4 | BCNU + standard RT vs BCNU + accelerated RT vs Cisplatin/BCNU + standard RT vs Cisplatin/BCNU + accelerated RT | NR | OS | No |
| 81 | 16 | AA, GBM, PNET | 50 | 4 | 2 | Vincristine + RT vs Vincristine/CCNU/procarbazine/ hydroxyurea/cisplatin/cytarabine/dacarbine/methylprednisolone + RT | NR | PFS | Yes |
| 82 | 130 | GBM | 36 | NR | 2 | TMZ + RT vs RT | 11 | OS, PFS | Yes |
| 83 | 712 | High grade gliomas | 37 | 53 | 2 | BCNU + Hyperfractionated RT vs BCNU + Standard RT | NR | OS | No |
| 84 | 48 | Medulloblastoma | 29 | 7 | 2 | Vincristine/etoposide/cisplatin + RT vs RT | NR | OS | No |
| 85 | 745 | High grade gliomas | 40 | 42 | 4 | RT vs RT 🡪 TMZ vs TMZ + RT vs TMZ + RT 🡪 TMZ | 27 | OS | Yes |

* if median age was not available, mean age was used.

Abbreviations: GTV, gross tumour volume; CTV, clinical tumour volume; vs, versus; NR, not reported; PFS, progression free survival; GBM, glioblastoma multiforme; OS, overall survival; EFS, event free survival; BCNU, carmustine; DIPG, diffuse intrinsic pontine glioma; PCV, procarbazine, lomustine, vincristine; AA, anaplastic astrocytoma; ACNU, nimustine; PCZ, procarbazine; AO, anaplastic oligoastrocytoma; EMD, cilengitide; TMZ, temozolomide; RT, radiotherapy; TTF, time to failure; PPX, paclitaxel poliglumex; VM26, teniposide; Ara-C, cytarabine; IFNβ, interferon-beta; BUdR, Bromodeoxyuridine; TTP, time to progression; CCDP, cisplatin; GCV, ganciclovir; DBD, dibromodulcitol; NU, nitrosurea;
