# Supplementary material for: Quality of reporting of cranial irradiation techniques in randomized controlled trials of primary brain tumors: A systematic review
Source: PLoS One. 2020 Nov 5;15(11):e0241566. doi: 10.1371/journal.pone.0241566 (PMC7644083; doi:10.1371/journal.pone.0241566)
Supplement: S4 Table — (DOCX) [file pone.0241566.s004.docx]

S4 Table. Risk of bias in the methodological quality

| Study | Risk of bias arising from the randomisation process | Risk of bias due to deviations from the intended interventions (effect of assignment to intervention) | Risk of bias due to deviations from the intended interventions (effect of adhering to intervention) | Risk of bias due to missing outcome data | Risk of bias in measurement of the outcome | Risk of bias in selection of the reported result | Overall risk of bias |
| --- | --- | --- | --- | --- | --- | --- | --- |
| 1 | Some concerns | Low risk | Low risk | Low risk | Some concerns | Low risk | Some concerns |
| 2 | Low risk | Low risk | Low risk | Low risk | Low risk | Low risk | Low risk |
| 3 | Low risk | Low risk | Low risk | Low risk | Low risk | Low risk | Low risk |
| 4 | Low risk | Low risk | Low risk | Low risk | Low risk | Low risk | Low risk |
| 5 | Low risk | Low risk | Low risk | Low risk | Low risk | Low risk | Low risk |
| 6 | Low risk | Low risk | Low risk | Low risk | Low risk | Low risk | Low risk |
| 7 | Low risk | Low risk | Some concerns | Low risk | Low risk | Low risk | Some concerns |
| 8 | Low risk | Low risk | Low risk | Low risk | Low risk | Low risk | Low risk |
| 9 | Low risk | Low risk | Low risk | Low risk | Low risk | Low risk | Low risk |
| 10 | Some concerns | Low risk | Low risk | Low risk | Low risk | Low risk | Some concerns |
| 11 | Some concerns | Some concerns | Low risk | Low risk | Low risk | Low risk | Some concerns |
| 12 | Low risk | Low risk | Low risk | Low risk | Low risk | Low risk | Low risk |
| 13 | Low risk | Low risk | Low risk | Low risk | Low risk | Low risk | Low risk |
| 14 | Low risk | Low risk | Low risk | Low risk | Low risk | Low risk | Low risk |
| 15 | Low risk | Low risk | Low risk | Low risk | Low risk | Low risk | Low risk |
| 16 | Low risk | Low risk | Low risk | Low risk | Low risk | Low risk | Low risk |
| 17 | Low risk | Some concerns | Low risk | Low risk | Low risk | Low risk | Some concerns |
| 18 | Some concerns | Low risk | Low risk | Low risk | Low risk | Low risk | Some concerns |
| 19 | Low risk | Some concerns | Some concerns | Low risk | Low risk | Low risk | Some concerns |
| 20 | Some concerns | Low risk | Low risk | Low risk | Low risk | Low risk | Some concerns |
| 21 | Low risk | Low risk | Low risk | Low risk | Low risk | Low risk | Low risk |
| 22 | Low risk | Low risk | Low risk | Low risk | Low risk | Low risk | Low risk |
| 23 | Low risk | Low risk | Low risk | Low risk | Low risk | Low risk | Low risk |
| 24 | Low risk | Low risk | Some concerns | Low risk | Low risk | Low risk | Some concerns |
| 25 | Low risk | Low risk | Some concerns | Low risk | Low risk | Low risk | Some concerns |
| 26 | Low risk | Low risk | Low risk | Low risk | Low risk | Low risk | Low risk |
| 27 | Low risk | Low risk | Low risk | Low risk | Low risk | Low risk | Low risk |
| 28 | Low risk | Low risk | Low risk | Low risk | Low risk | Low risk | Low risk |
| 29 | Some concerns | Low risk | Low risk | Low risk | Low risk | Low risk | Some concerns |
| 30 | Low risk | Low risk | Low risk | Low risk | Low risk | Low risk | Low risk |
| 31 | Low risk | Some concerns | Some concerns | Low risk | Low risk | Low risk | Some concerns |
| 32 | Low risk | Some concerns | Some concerns | Low risk | Low risk | Low risk | Some concerns |
| 33 | Low risk | Low risk | Low risk | Low risk | Low risk | Low risk | Low risk |
| 34 | Some concerns | Low risk | Low risk | Low risk | Low risk | Low risk | Low risk |
| 35 | Low risk | Low risk | Low risk | Low risk | Low risk | Low risk | Low risk |
| 36 | Low risk | Low risk | Low risk | Low risk | Low risk | Low risk | Low risk |
| 37 | Low risk | Low risk | Low risk | Low risk | Low risk | Low risk | Low risk |
| 38 | Low risk | Low risk | Low risk | Low risk | Low risk | Low risk | Low risk |
| 39 | Low risk | Low risk | Low risk | Low risk | Low risk | Low risk | Low risk |
| 40 | Low risk | Some concerns | Some concerns | Low risk | Low risk | Low risk | Some concerns |
| 41 | Some concerns | Low risk | Low risk | Low risk | Low risk | Low risk | Some concerns |
| 42 | Low risk | Low risk | Low risk | Low risk | Low risk | Low risk | Low risk |
| 43 | Low risk | Low risk | Low risk | Low risk | Low risk | Low risk | Low risk |
| 44 | Low risk | Low risk | Low risk | Low risk | Low risk | Low risk | Low risk |
| 45 | Low risk | Low risk | Low risk | Low risk | Low risk | Low risk | Low risk |
| 46 | Low risk | Low risk | Low risk | Low risk | Low risk | Low risk | Low risk |
| 47 | Low risk | Low risk | Low risk | Low risk | Low risk | Low risk | Low risk |
| 48 | Low risk | Low risk | Low risk | Low risk | Low risk | Low risk | Low risk |
| 49 | Some concerns | Some concerns | Some concerns | Some concerns | Some concerns | Some concerns | Some concerns |
| 50 | Low risk | Low risk | Low risk | Low risk | Low risk | Low risk | Low risk |
| 51 | Some concerns | Low risk | Low risk | Low risk | Low risk | Low risk | Some concerns |
| 52 | Low risk | Low risk | Low risk | Low risk | Low risk | Low risk | Low risk |
| 53 | Low risk | Low risk | Low risk | Low risk | Low risk | Low risk | Low risk |
| 54 | Some concerns | Low risk | Low risk | Low risk | Low risk | Low risk | Some concerns |
| 55 | Low risk | Low risk | Low risk | Low risk | Low risk | Low risk | Low risk |
| 56 | Low risk | Low risk | Low risk | Low risk | Low risk | Low risk | Low risk |
| 57 | Some concerns | Low risk | Low risk | Low risk | Low risk | Low risk | Some concerns |
| 58 | Low risk | Low risk | Low risk | Low risk | Low risk | Low risk | Low risk |
| 59 | Low risk | Low risk | Low risk | Low risk | Low risk | Low risk | Low risk |
| 60 | Low risk | Low risk | Low risk | Low risk | Low risk | Low risk | Low risk |
| 61 | Low risk | Some concerns | Some concerns | Low risk | Low risk | Low risk | Some concerns |
| 62 | Low risk | Low risk | Low risk | Low risk | Low risk | Low risk | Low risk |
| 63 | Low risk | Low risk | Low risk | Low risk | Low risk | Low risk | Low risk |
| 64 | Low risk | Low risk | Low risk | Low risk | Low risk | Low risk | Low risk |
| 65 | Low risk | Low risk | Low risk | Low risk | Low risk | Low risk | Low risk |
| 66 | Low risk | Low risk | Low risk | Low risk | Low risk | Low risk | Low risk |
| 67 | Low risk | Low risk | Low risk | Low risk | Low risk | Low risk | Low risk |
| 68 | Low risk | Low risk | Low risk | Low risk | Low risk | Low risk | Low risk |
| 69 | Some concerns | Low risk | Low risk | Low risk | Low risk | Low risk | Some concerns |
| 70 | Low risk | Low risk | Low risk | Low risk | Low risk | Low risk | Low risk |
| 71 | Low risk | Low risk | Low risk | Low risk | Low risk | Low risk | Low risk |
| 72 | Low risk | Low risk | Low risk | Low risk | Low risk | Low risk | Low risk |
| 73 | Low risk | Low risk | Low risk | Low risk | Low risk | Low risk | Low risk |
| 74 | Low risk | Low risk | Low risk | Low risk | Low risk | Low risk | Low risk |
| 75 | Low risk | Some concerns | Low risk | Low risk | Low risk | Low risk | Some concerns |
| 76 | Low risk | Low risk | Low risk | Low risk | Low risk | Low risk | Low risk |
| 77 | Low risk | Low risk | Low risk | Low risk | Low risk | Low risk | Low risk |
| 78 | Low risk | Low risk | Low risk | Low risk | Low risk | Low risk | Low risk |
| 79 | Low risk | Low risk | Low risk | Low risk | Low risk | Low risk | Low risk |
| 80 | Low risk | Low risk | Low risk | Low risk | Low risk | Low risk | Low risk |
| 81 | Some concerns | Low risk | Low risk | Low risk | Low risk | Low risk | Some concerns |
| 82 | Low risk | Low risk | Low risk | Low risk | Low risk | Low risk | Low risk |
| 83 | Low risk | Low risk | Low risk | Low risk | Low risk | Low risk | Low risk |
| 84 | Some concerns | Some concerns | Low risk | Low risk | Low risk | Some concerns | Some concerns |
| 85 | Low risk | Low risk | Low risk | Low risk | Low risk | Low risk | Low risk |
