# Supplementary material for: Quality of reporting of cranial irradiation techniques in randomized controlled trials of primary brain tumors: A systematic review
Source: PLoS One. 2020 Nov 5;15(11):e0241566. doi: 10.1371/journal.pone.0241566 (PMC7644083; doi:10.1371/journal.pone.0241566)
Supplement: S5 Table — (DOCX) [file pone.0241566.s005.docx]

S5 Table. Adequate reporting of each criterion

| Study | Radiotherapy dose prescription method | Radiotherapy dose planning procedures | Organ at risk dose constraints | Target volume definition | Immobilization procedures | Treatment verification procedures | Total radiation dose | Fractionation schedule | Conduct of quality assurance | Deviation in the radiation treatment planning and delivery | Total number of criterion which were reported adequately | Adequate quality in reporting |
| --- | --- | --- | --- | --- | --- | --- | --- | --- | --- | --- | --- | --- |
| 1 | Not reported adequately | Reported adequately | Not reported adequately | Reported adequately | Reported adequately | Not reported adequately | Reported adequately | Not reported adequately | Not reported adequately | Not reported adequately | 4 | No |
| 2 | Reported adequately | Reported adequately | Reported adequately | Reported adequately | Reported adequately | Reported adequately | Reported adequately | Reported adequately | Reported adequately | Not reported adequately | 9 | Yes |
| 3 | Reported adequately | Reported adequately | Not reported adequately | Reported adequately | Not reported adequately | Not reported adequately | Reported adequately | Reported adequately | Not reported adequately | Not reported adequately | 5 | No |
| 4 | Not reported adequately | Reported adequately | Reported adequately | Reported adequately | Reported adequately | Not reported adequately | Reported adequately | Reported adequately | Not reported adequately | Not reported adequately | 6 | No |
| 5 | Reported adequately | Reported adequately | Not reported adequately | Reported adequately | Not reported adequately | Not reported adequately | Reported adequately | Reported adequately | Reported adequately | Reported adequately | 7 | Yes |
| 6 | Not reported adequately | Not reported adequately | Not reported adequately | Reported adequately | Reported adequately | Reported adequately | Reported adequately | Reported adequately | Not reported adequately | Not reported adequately | 5 | No |
| 7 | Not reported adequately | Reported adequately | Reported adequately | Reported adequately | Not reported adequately | Not reported adequately | Reported adequately | Reported adequately | Not reported adequately | Not reported adequately | 5 | No |
| 8 | Reported adequately | Reported adequately | Reported adequately | Reported adequately | Not reported adequately | Reported adequately | Reported adequately | Reported adequately | Reported adequately | Reported adequately | 9 | Yes |
| 9 | Reported adequately | Reported adequately | Not reported adequately | Reported adequately | Reported adequately | Reported adequately | Reported adequately | Reported adequately | Reported adequately | Reported adequately | 9 | Yes |
| 10 | Not reported adequately | Not reported adequately | Not reported adequately | Reported adequately | Not reported adequately | Not reported adequately | Reported adequately | Reported adequately | Not reported adequately | Not reported adequately | 3 | No |
| 11 | Not reported adequately | Reported adequately | Reported adequately | Reported adequately | Reported adequately | Not reported adequately | Reported adequately | Not reported adequately | Reported adequately | Not reported adequately | 6 | No |
| 12 | Reported adequately | Not reported adequately | Reported adequately | Reported adequately | Not reported adequately | Reported adequately | Reported adequately | Reported adequately | Reported adequately | Reported adequately | 8 | Yes |
| 13 | Not reported adequately | Not reported adequately | Not reported adequately | Not reported adequately | Not reported adequately | Not reported adequately | Reported adequately | Reported adequately | Not reported adequately | Not reported adequately | 2 | No |
| 14 | Reported adequately | Reported adequately | Reported adequately | Reported adequately | Reported adequately | Reported adequately | Reported adequately | Reported adequately | Reported adequately | Reported adequately | 10 | Yes |
| 15 | Not reported adequately | Not reported adequately | Not reported adequately | Not reported adequately | Not reported adequately | Not reported adequately | Reported adequately | Reported adequately | Not reported adequately | Not reported adequately | 2 | No |
| 16 | Not reported adequately | Not reported adequately | Not reported adequately | Reported adequately | Not reported adequately | Not reported adequately | Reported adequately | Reported adequately | Not reported adequately | Not reported adequately | 3 | No |
| 17 | Not reported adequately | Reported adequately | Not reported adequately | Not reported adequately | Not reported adequately | Not reported adequately | Reported adequately | Reported adequately | Not reported adequately | Not reported adequately | 3 | No |
| 18 | Reported adequately | Not reported adequately | Reported adequately | Reported adequately | Reported adequately | Not reported adequately | Reported adequately | Reported adequately | Not reported adequately | Not reported adequately | 6 | No |
| 19 | Not reported adequately | Not reported adequately | Not reported adequately | Not reported adequately | Not reported adequately | Not reported adequately | Reported adequately | Not reported adequately | Not reported adequately | Not reported adequately | 1 | No |
| 20 | Not reported adequately | Not reported adequately | Not reported adequately | Reported adequately | Reported adequately | Not reported adequately | Reported adequately | Reported adequately | Not reported adequately | Not reported adequately | 4 | No |
| 21 | Reported adequately | Reported adequately | Reported adequately | Reported adequately | Reported adequately | Reported adequately | Reported adequately | Reported adequately | Reported adequately | Reported adequately | 10 | Yes |
| 22 | Not reported adequately | Reported adequately | Not reported adequately | Reported adequately | Not reported adequately | Not reported adequately | Reported adequately | Reported adequately | Not reported adequately | Not reported adequately | 4 | No |
| 23 | Not reported adequately | Not reported adequately | Not reported adequately | Reported adequately | Not reported adequately | Not reported adequately | Reported adequately | Reported adequately | Not reported adequately | Not reported adequately | 3 | No |
| 24 | Not reported adequately | Not reported adequately | Not reported adequately | Reported adequately | Not reported adequately | Not reported adequately | Reported adequately | Reported adequately | Not reported adequately | Not reported adequately | 3 | No |
| 25 | Not reported adequately | Not reported adequately | Not reported adequately | Not reported adequately | Not reported adequately | Not reported adequately | Not reported adequately | Reported adequately | Not reported adequately | Not reported adequately | 1 | No |
| 26 | Reported adequately | Reported adequately | Reported adequately | Reported adequately | Reported adequately | Reported adequately | Reported adequately | Reported adequately | Reported adequately | Reported adequately | 10 | Yes |
| 27 | Reported adequately | Not reported adequately | Not reported adequately | Reported adequately | Not reported adequately | Not reported adequately | Reported adequately | Reported adequately | Reported adequately | Reported adequately | 6 | No |
| 28 | Reported adequately | Reported adequately | Reported adequately | Reported adequately | Not reported adequately | Not reported adequately | Reported adequately | Reported adequately | Not reported adequately | Not reported adequately | 6 | No |
| 29 | Reported adequately | Reported adequately | Not reported adequately | Reported adequately | Reported adequately | Reported adequately | Reported adequately | Reported adequately | Not reported adequately | Not reported adequately | 7 | Yes |
| 30 | Reported adequately | Not reported adequately | Not reported adequately | Reported adequately | Reported adequately | Reported adequately | Reported adequately | Reported adequately | Reported adequately | Reported adequately | 8 | Yes |
| 31 | Not reported adequately | Not reported adequately | Not reported adequately | Not reported adequately | Not reported adequately | Not reported adequately | Reported adequately | Reported adequately | Reported adequately | Not reported adequately | 3 | No |
| 32 | Reported adequately | Not reported adequately | Not reported adequately | Reported adequately | Not reported adequately | Reported adequately | Reported adequately | Reported adequately | Reported adequately | Not reported adequately | 6 | No |
| 33 | Not reported adequately | Not reported adequately | Not reported adequately | Reported adequately | Not reported adequately | Reported adequately | Reported adequately | Reported adequately | Reported adequately | Not reported adequately | 5 | No |
| 34 | Not reported adequately | Not reported adequately | Not reported adequately | Reported adequately | Not reported adequately | Not reported adequately | Reported adequately | Reported adequately | Not reported adequately | Not reported adequately | 3 | No |
| 35 | Reported adequately | Reported adequately | Not reported adequately | Reported adequately | Reported adequately | Reported adequately | Reported adequately | Reported adequately | Reported adequately | Reported adequately | 9 | Yes |
| 36 | Not reported adequately | Not reported adequately | Not reported adequately | Reported adequately | Not reported adequately | Not reported adequately | Reported adequately | Reported adequately | Not reported adequately | Not reported adequately | 3 | No |
| 37 | Reported adequately | Reported adequately | Not reported adequately | Reported adequately | Not reported adequately | Not reported adequately | Reported adequately | Reported adequately | Reported adequately | Reported adequately | 7 | Yes |
| 38 | Reported adequately | Reported adequately | Reported adequately | Reported adequately | Reported adequately | Reported adequately | Reported adequately | Reported adequately | Reported adequately | Not reported adequately | 9 | Yes |
| 39 | Not reported adequately | Not reported adequately | Not reported adequately | Not reported adequately | Not reported adequately | Not reported adequately | Reported adequately | Reported adequately | Reported adequately | Not reported adequately | 3 | No |
| 40 | Not reported adequately | Not reported adequately | Not reported adequately | Not reported adequately | Not reported adequately | Not reported adequately | Reported adequately | Reported adequately | Not reported adequately | Not reported adequately | 2 | No |
| 41 | Not reported adequately | Not reported adequately | Not reported adequately | Not reported adequately | Not reported adequately | Not reported adequately | Reported adequately | Reported adequately | Not reported adequately | Not reported adequately | 2 | No |
| 42 | Not reported adequately | Not reported adequately | Not reported adequately | Not reported adequately | Not reported adequately | Not reported adequately | Reported adequately | Reported adequately | Not reported adequately | Not reported adequately | 2 | No |
| 43 | Not reported adequately | Not reported adequately | Not reported adequately | Reported adequately | Not reported adequately | Not reported adequately | Reported adequately | Reported adequately | Not reported adequately | Not reported adequately | 3 | No |
| 44 | Reported adequately | Reported adequately | Reported adequately | Reported adequately | Reported adequately | Reported adequately | Reported adequately | Reported adequately | Reported adequately | Not reported adequately | 9 | Yes |
| 45 | Not reported adequately | Not reported adequately | Not reported adequately | Not reported adequately | Not reported adequately | Not reported adequately | Not reported adequately | Not reported adequately | Not reported adequately | Not reported adequately | 0 | No |
| 46 | Not reported adequately | Not reported adequately | Not reported adequately | Not reported adequately | Not reported adequately | Not reported adequately | Reported adequately | Reported adequately | Not reported adequately | Not reported adequately | 2 | No |
| 47 | Not reported adequately | Not reported adequately | Not reported adequately | Reported adequately | Not reported adequately | Not reported adequately | Reported adequately | Reported adequately | Not reported adequately | Not reported adequately | 3 | No |
| 48 | Not reported adequately | Not reported adequately | Not reported adequately | Reported adequately | Reported adequately | Not reported adequately | Reported adequately | Reported adequately | Not reported adequately | Not reported adequately | 4 | No |
| 49 | Not reported adequately | Not reported adequately | Not reported adequately | Not reported adequately | Not reported adequately | Not reported adequately | Reported adequately | Not reported adequately | Not reported adequately | Not reported adequately | 1 | No |
| 50 | Not reported adequately | Not reported adequately | Not reported adequately | Reported adequately | Not reported adequately | Not reported adequately | Reported adequately | Reported adequately | Not reported adequately | Not reported adequately | 3 | No |
| 51 | Not reported adequately | Not reported adequately | Not reported adequately | Not reported adequately | Not reported adequately | Not reported adequately | Reported adequately | Reported adequately | Not reported adequately | Not reported adequately | 2 | No |
| 52 | Not reported adequately | Not reported adequately | Not reported adequately | Reported adequately | Not reported adequately | Not reported adequately | Reported adequately | Reported adequately | Not reported adequately | Not reported adequately | 3 | No |
| 53 | Not reported adequately | Not reported adequately | Not reported adequately | Reported adequately | Not reported adequately | Not reported adequately | Reported adequately | Reported adequately | Reported adequately | Not reported adequately | 4 | No |
| 54 | Not reported adequately | Not reported adequately | Not reported adequately | Reported adequately | Not reported adequately | Not reported adequately | Reported adequately | Reported adequately | Not reported adequately | Not reported adequately | 3 | No |
| 55 | Reported adequately | Not reported adequately | Not reported adequately | Reported adequately | Not reported adequately | Not reported adequately | Reported adequately | Reported adequately | Not reported adequately | Not reported adequately | 4 | No |
| 56 | Not reported adequately | Not reported adequately | Not reported adequately | Not reported adequately | Not reported adequately | Not reported adequately | Reported adequately | Reported adequately | Not reported adequately | Not reported adequately | 2 | No |
| 57 | Not reported adequately | Not reported adequately | Not reported adequately | Reported adequately | Not reported adequately | Not reported adequately | Reported adequately | Reported adequately | Not reported adequately | Not reported adequately | 3 | No |
| 58 | Not reported adequately | Not reported adequately | Not reported adequately | Reported adequately | Reported adequately | Not reported adequately | Reported adequately | Reported adequately | Not reported adequately | Not reported adequately | 4 | No |
| 59 | Not reported adequately | Not reported adequately | Not reported adequately | Reported adequately | Not reported adequately | Not reported adequately | Reported adequately | Reported adequately | Not reported adequately | Not reported adequately | 3 | No |
| 60 | Reported adequately | Reported adequately | Reported adequately | Reported adequately | Reported adequately | Reported adequately | Reported adequately | Reported adequately | Reported adequately | Not reported adequately | 9 | Yes |
| 61 | Not reported adequately | Not reported adequately | Not reported adequately | Not reported adequately | Not reported adequately | Not reported adequately | Reported adequately | Reported adequately | Not reported adequately | Not reported adequately | 2 | No |
| 62 | Not reported adequately | Not reported adequately | Not reported adequately | Reported adequately | Not reported adequately | Not reported adequately | Reported adequately | Reported adequately | Not reported adequately | Not reported adequately | 3 | No |
| 63 | Not reported adequately | Not reported adequately | Not reported adequately | Not reported adequately | Not reported adequately | Not reported adequately | Reported adequately | Reported adequately | Not reported adequately | Not reported adequately | 2 | No |
| 64 | Not reported adequately | Not reported adequately | Not reported adequately | Reported adequately | Not reported adequately | Not reported adequately | Reported adequately | Reported adequately | Not reported adequately | Not reported adequately | 3 | No |
| 65 | Reported adequately | Reported adequately | Reported adequately | Reported adequately | Reported adequately | Reported adequately | Reported adequately | Reported adequately | Not reported adequately | Not reported adequately | 8 | Yes |
| 66 | Reported adequately | Reported adequately | Reported adequately | Reported adequately | Reported adequately | Reported adequately | Reported adequately | Reported adequately | Reported adequately | Not reported adequately | 9 | Yes |
| 67 | Not reported adequately | Not reported adequately | Not reported adequately | Not reported adequately | Not reported adequately | Not reported adequately | Reported adequately | Reported adequately | Not reported adequately | Not reported adequately | 2 | No |
| 68 | Not reported adequately | Not reported adequately | Not reported adequately | Reported adequately | Not reported adequately | Not reported adequately | Reported adequately | Reported adequately | Not reported adequately | Not reported adequately | 3 | No |
| 69 | Not reported adequately | Not reported adequately | Not reported adequately | Reported adequately | Not reported adequately | Not reported adequately | Reported adequately | Reported adequately | Not reported adequately | Not reported adequately | 3 | No |
| 70 | Not reported adequately | Not reported adequately | Not reported adequately | Reported adequately | Not reported adequately | Not reported adequately | Reported adequately | Reported adequately | Not reported adequately | Not reported adequately | 3 | No |
| 71 | Not reported adequately | Not reported adequately | Not reported adequately | Not reported adequately | Not reported adequately | Not reported adequately | Reported adequately | Reported adequately | Not reported adequately | Not reported adequately | 2 | No |
| 72 | Reported adequately | Reported adequately | Not reported adequately | Reported adequately | Reported adequately | Not reported adequately | Reported adequately | Reported adequately | Reported adequately | Not reported adequately | 7 | Yes |
| 73 | Not reported adequately | Not reported adequately | Not reported adequately | Not reported adequately | Not reported adequately | Not reported adequately | Reported adequately | Not reported adequately | Not reported adequately | Not reported adequately | 1 | No |
| 74 | Not reported adequately | Not reported adequately | Not reported adequately | Reported adequately | Not reported adequately | Not reported adequately | Reported adequately | Reported adequately | Not reported adequately | Not reported adequately | 3 | No |
| 75 | Not reported adequately | Not reported adequately | Not reported adequately | Reported adequately | Not reported adequately | Not reported adequately | Reported adequately | Reported adequately | Reported adequately | Not reported adequately | 4 | No |
| 76 | Not reported adequately | Not reported adequately | Not reported adequately | Reported adequately | Reported adequately | Not reported adequately | Reported adequately | Reported adequately | Not reported adequately | Not reported adequately | 4 | No |
| 77 | Reported adequately | Reported adequately | Reported adequately | Reported adequately | Reported adequately | Reported adequately | Reported adequately | Reported adequately | Reported adequately | Not reported adequately | 9 | Yes |
| 78 | Reported adequately | Reported adequately | Reported adequately | Reported adequately | Reported adequately | Reported adequately | Reported adequately | Reported adequately | Reported adequately | Reported adequately | 10 | Yes |
| 79 | Reported adequately | Reported adequately | Not reported adequately | Reported adequately | Not reported adequately | Reported adequately | Reported adequately | Reported adequately | Reported adequately | Reported adequately | 8 | Yes |
| 80 | Not reported adequately | Not reported adequately | Not reported adequately | Not reported adequately | Not reported adequately | Not reported adequately | Reported adequately | Reported adequately | Not reported adequately | Not reported adequately | 2 | No |
| 81 | Reported adequately | Not reported adequately | Not reported adequately | Reported adequately | Not reported adequately | Reported adequately | Reported adequately | Reported adequately | Reported adequately | Not reported adequately | 6 | No |
| 82 | Not reported adequately | Reported adequately | Not reported adequately | Reported adequately | Reported adequately | Not reported adequately | Reported adequately | Reported adequately | Not reported adequately | Not reported adequately | 5 | No |
| 83 | Not reported adequately | Reported adequately | Reported adequately | Reported adequately | Not reported adequately | Reported adequately | Reported adequately | Reported adequately | Reported adequately | Reported adequately | 8 | Yes |
| 84 | Not reported adequately | Not reported adequately | Not reported adequately | Reported adequately | Not reported adequately | Not reported adequately | Reported adequately | Reported adequately | Not reported adequately | Not reported adequately | 3 | No |
| 85 | Reported adequately | Reported adequately | Reported adequately | Reported adequately | Reported adequately | Reported adequately | Reported adequately | Reported adequately | Reported adequately | Reported adequately | 10 | Yes |
