# Supplementary material for: Quality of reporting of cranial irradiation techniques in randomized controlled trials of primary brain tumors: A systematic review
Source: PLoS One. 2020 Nov 5;15(11):e0241566. doi: 10.1371/journal.pone.0241566 (PMC7644083; doi:10.1371/journal.pone.0241566)
Supplement: Attachment — Submitted filename: Response to reviewers.docx [file pone.0241566.s007.docx]

Response to reviewers

| No. | Reviewer | Reviewer comments | Our response |
| --- | --- | --- | --- |
| 1 | 1 | This meta-analysis is about "Quality of reporting of cranial irradiation techniques in randomized controlled trials of primary brain tumors: a systematic review". In my opinion, the article does not have the necessary quality to be accepted | We have improved the quality of manuscript according to the other reviewers. Please reconsider your decision on our manuscript. |
| 2 | 2 | Introduction: What is the rationale for determining predictors for the quality of reporting? How will this be used? This should be described in the introduction | Thank you for the comments.  The rationale for determining the predictors for adequate quality of reporting of cranial radiation therapy technique is to help readers understand what are the characteristics of a trial report that are predictive of adequate quality in reporting of cranial radiation therapy technique. This may help the readers to be more confident in the results reported by the trial investigators if there is adequate quality clear idea on how the radiation therapy treatment is delivered.  We have included the following paragraph in the introduction section. “Several studies have suggested that the quality of reporting of RT treatment were inconsistent across randomized trials of lymphoma, head and neck, prostate and lung cancers[4-7]. However, the evidence on the quality of reporting of cranial irradiation (CR) technique in randomized trials of primary brain tumors and factors that may predict the quality of reporting is unknown. It is helpful for the readers to know what are the characteristics of a trial report that are predictive for adequate quality in reporting of cranial RT technique. This will help readers to be more confident in the results reported by the trial investigators if there is a clear understanding on how the radiation therapy treatment is delivered.” |
| 3 |  | Method: Were only studies with malignant primary brain tumors included? Or also benign tumors? Also, it should be described if the focus is on pediatric or adult patients, or both? In short, the specific in- and exclusion criteria should be reported. | Thank you for the comments.  We only included studies with malignant tumours. We focused on both paediatric and adult patients.  We have elaborated on the inclusion and exclusion criteria of studies in the methods section. “This study included full publication of randomized trials of pediatric or adult patients with histologically or radiologically proven malignant primary brain tumors. Trials that include brain metastases were excluded as they were not primary brain tumors. Trials that include primary central nervous system lymphoma were specifically excluded as the cranial irradiation technique that are used in the treatment of primary central nervous system lymphoma is the standard whole brain radiation therapy technique that is well described in standard radiation oncology textbook and it is unlikely that the trial investigators will describe the standard whole brain radiation therapy technique in detail in their trial reports.” |
| 4 |  | Methods: What was the reason to exclude PCNSL? I understand that brain metastases are excluded, as they are not primary brain tumors, but it is unclear why studies including PCNSL patients are excluded. | We excluded trials that include primary central nervous system lymphoma as the cranial irradiation technique that are used in the treatment of primary central nervous system lymphoma is the standard whole brain radiation therapy technique that is well described in standard radiation oncology textbook and it is unlikely that the trial investigators will describe the standard whole brain radiation therapy technique in detail in their trial reports. |
|  |  | Methods: Why were only MeSH terms included in the search strategy? Also, only two databases were searched. This should be recognized as a limitation. | Thank you for the comments.  We apologize for the confusion. As reported in the S1 Table in the supplementary materials, our search strategy included MESH terms for brain neoplasms and radiation therapy as well as its synonyms to be searched as keywords in title and abstract. We have further clarified our search strategy in the main text as “The search strategy included the medical subject headings (MESH) terms and its synonyms for “brain neoplasms and “radiotherapy”. The synonyms were searched as key words in the titles and abstracts. The search was limited to randomized trials and time period from January 1999 to November 2019”.  We agree that limiting the search to two databases is a limitation. We have acknowledged this as a limitation in the discussion section “Fourthly, we searched only two databases mainly MEDLINE via PubMed and EMBASE for eligible studies. It is possible that our search is not sufficiently comprehensive and some eligible studies may have been missed.” |
|  |  | Methods: The authors state that the 10 quality criteria have been described in previous publications. What are the references? And were these 10 criteria also used in the previous reviews (as described in the introduction) to assess the quality of reporting of irradiation techniques? What were similarities and differences? And if not equal, why were different methods used? | We apologized for the confusion.  We have included the references as suggested.  These exact 10 criteria were used in previous reviews and current study to assess the quality of reporting of irradiation techniques. For assessment of quality of thoracic radiation techniques, the authors used an extra criteria on whether the algorithm was used to correct for tissue inhomogeneity. We did not use this criteria as it is not applicable in cranial radiation therapy treatment. The tissue inhomogeneity correction is required to correct the doses for air and lung tissue interfaces for lung radiation treatment. |
|  |  | Methods: Was each variable first tested in a univariable model before entering in the multivariable model? If not, this should be explained. | We did not test each variable in a univariable model. We employed a backward stepwise regression model approach where all the explanatory variables are first entered in the model and at each step, the explanatory variables were gradually eliminated from the regression model when its p value is more than 0.2 to find the reduced model that best explains the data. We prefer this backward stepwise approach because it reduces the number of predictors and may help to reduce multi-collinearity problem and resolve overfitting.  We have elaborated in the methods section “We used a backward stepwise multivariable logistic regression model approach to identify the predictors and its associated odds ratio for adequate quality in reporting of CR technique. This approach allows all the possible explanatory predictors to be first entered in the model and at each step, we allowed the explanatory predictors to be gradually eliminated from the regression model when its p value is more than 0.2 to find the reduced model that best explains the data. Predictors with p value less than 0.05 in the multivariable logistic regression were considered statistically significant. We prefer this backward stepwise approach because it reduces the number of predictors and may help to reduce multi-collinearity problem and resolve overfitting. All potential predictors except for year of publication, sample size and impact factor of the journal for the year of publication were analysed as categorical variables. All statistical analysis was performed using STATA (version16.0, StataCorp).” |
|  |  | Methods: What is the rationale for including ‘bias in reporting the primary efficacy endpoint’ or ‘bias in reporting of primary toxicity endpoint’ as predictors for good quality of reporting of RT techniques? And not characteristics such as tumor type? | We apologize for the confusion. After further discussion within the study team, we agree that there is no strong plausible rationale to explain the relationship between bias in reporting of primary efficacy and toxicity endpoints. We have removed these two variables.  We have included primary brain tumor histology (high grade glioma versus others) as suggested as one of the predictors for adequate quality of reporting of RT techniques. |
|  |  | Methods: only 23 studies were classified as ‘adequate quality in reporting’. This means that only 2 (and max 3) variables can be included in the multivariable model, otherwise it would be overfitted. In this study, more than 3 variables seem to be included, limiting the reliability of the model. | We agree with the suggestions. Based on the updated backward stepwise multivariable logistic regression analysis, we have managed to identify three variables with p values less than 0.05 in the multivariable logistic regression model. |
|  |  | Results: Data on each eligible study was collected, including characteristics such as patient population. It would be helpful to add characteristics (tumor type, number of patients, intervention, sociodemographics) to the supplemental table. | We have added a table of study characteristics in the supplementary materials on patient population, sample size, intervention, primary outcome and whether the prespecified endpoint was met. |
|  |  | Results: Bias in reporting the primary efficacy endpoint was predictive of good quality reporting of RT techniques? This is counterintuitive: if they would not properly report the primary endpoints, why would they properly report information on RT techniques? I am not convinced by the explanation provided by the authors. | We have removed the bias in reporting of primary efficacy endpoint. |
|  |  | Results: The odds ratios 6.67 and 5.17 are converted to percentages. However, shouldn’t these be 567% and 417% instead of 667% and 517% (and OR of 2 is a 100% increase in chance, not 200%)? | We have rephrased the updated odds ratio in the results section as  “The odds of having adequate quality in reporting of CR technique among trials conducted by cooperative group were 4.65 times that of non-cooperative group trials (odds ratio (OR) 4.65, 95% confidence interval (CI) 1.13 to 19.11, P value (P) = 0.033). The odds of having adequate quality in reporting of CR technique for trials that published their QA results were 8.5 times that of trials that did not publish their QA results (OR 8.50, 95% CI 1.87 to 38.56, P = 0.006). The odds of having adequate quality in reporting of CR technique among trials with some concerns in the methodological quality is 0.10 times that of trials with low risk of bias in the methodological quality (OR 0.10, 95% CI 0.01 to 0.81, P = 0.031).” |
|  |  | Discussion: The authors argue in the introduction that information on the radiation techniques should be reported adequately in RCTs, to reduce the risk of deviation in daily clinical practice. In this review, all type of primary brain tumors are combined. I wonder, however, if there are differences in trials for different types of brain tumors? And what about trials that were practice-changing versus those who were not? It would be informative if these subanalysis would be performed. | We have performed the multivariable logistic regression including the type of primary brain tumor and whether the trial met is pre-specified endpoint as predictors for adequate quality of reporting. They were eventually not included in the multivariable logistic model as their P values were more than 0.05.  We believed that the definition of practice-changing trials may be subjective. Hence we decided to use the definition of whether the trials met its pre-specified primary endpoint instead. |
|  |  | Discussion: as a limitation, a small sample size is mentioned, and therefore no definitive conclusion can be drawn. A definitive conclusion on what? | We have further clarified the statement by amending it to “Firstly, the sample size is fairly small, hence we are unable to have a definitive conclusion that the quality of reporting of CR technique is variable and suboptimal” |
|  | 3 | Specify study characteristics (e.g., PICOS, length of follow-up) and report characteristics (e.g., years considered, language, publication status) used as criteria for eligibility, giving rationale. | We have elaborated on the inclusion and exclusion criteria of studies in the methods section. “This study included full publication of randomized trials of paediatric or adult patients with histologically or radiologically proven malignant primary brain tumours. Trials that include brain metastases were excluded as they were not primary brain tumours. Trials that include primary central nervous system lymphoma were specifically excluded as the cranial irradiation technique that are used in the treatment of primary central nervous system lymphoma is the standard whole brain radiation therapy technique that is well described in standard radiation oncology textbook and it is unlikely that the trial investigators will describe the standard whole brain radiation therapy technique in detail in their trial reports.”  We have also provided a table (S3 table) summarizing the characteristics of included studies in the supplementary materials. |
|  |  | It should be need to present full electronic search strategy for at least one database, including any limits used, such that it could be repeated. | We have presented the full electronic search strategy in the supplementary material under S1 table. |
|  |  | Why the authors didn't use other major database such as ISI, Scopus? | We believe that PubMed and EMBASE are two important comprehensive medical databases for randomized trials and searching other major databases such as ISI and Scopus is unlikely to yield new eligible studies. Nevertheless we recognised limiting the search to two databases is a potential limitation and highlighted that in the discussion section as per reviewer 2 suggestion.  We have acknowledged this as a limitation in the discussion section “Fourthly, we searched only two databases mainly MEDLINE via PubMed and EMBASE for eligible studies. It is possible that our search is not sufficiently comprehensive and some eligible studies may have been missed.” |
|  |  | The full text article of any trial that appeared to meet the inclusion criteria was retrieved for closer inspection. Disagreements were resolved by consensus. If the agreement rate was assessed? Was the KAPPA statistics? | We did not assess the agreement rate quantitatively. We had a qualitative discussion between the reviewers and we have reached a consensus on every initial disagreements. |
|  |  | Describe methods used for assessing risk of bias of individual studies (including specification of whether this was done at the study or outcome level), and how this information is to be used in any data synthesis | We employed the Cochrane Risk of Bias tool (RoB2) to assess the risk of bias in the methodological quality of each study. We found that the risk of bias is a significant predictor of adequate quality reporting (The odds of having adequate quality in reporting of CR technique among trials with some concerns in the methodological quality is 0.10 times that of trials with low risk of bias in the methodological quality (OR 0.10, 95% CI 0.01 to 0.81, P = 0.031). We have updated the methods, results and discussion sections accordingly. |
|  |  | Describe the result of assessing risk of bias of individual studies? | We have included a table on the risk of bias in the methodological quality in the supplementary materials.  We have also updated the methods and results sections accordingly. |
|  |  | Table 4: p-values must be reported as three digit. | We have reported the p values as three digit as suggested. |

Other journal requirements

|  | Journal requirements | Our response |
| --- | --- | --- |
|  | Please ensure that your manuscript meets PLOS ONE's style requirements, including those for file naming. The PLOS ONE style templates can be found at  <https://journals.plos.org/plosone/s/file?id=wjVg/PLOSOne_formatting_sample_main_body.pdf> and  <https://journals.plos.org/plosone/s/file?id=ba62/PLOSOne_formatting_sample_title_authors_affiliations.pdf> | We have adjusted the manuscript to the Plos One style requirements |
|  | Upon revision, please ensure that inclusion and exclusion criteria for the selected studies are clearly stated in the Methods section. Please also give a rationale for using 7 criteria as a cut-off for good reporting of CR technique. Please also list which criteria were met for each study considered. | We have elaborated the inclusion and exclusion criteria of the included studies in the methods section.  We have highlighted in the methods that the 7 criteria is an arbitrary cut-off for adequate quality of reporting of CR technique.  We have included a table in the supplementary materials on which criteria were met for each study. |
|  | We noticed you have some minor occurrence(s) of overlapping text with the following previous publication(s), which needs to be addressed: <https://doi.org/10.1016/j.ijrobp.2017.06.862> <https://doi.org/10.1097/MD.0000000000016124> <https://doi.org/10.1200/JCO.2008.19.4522>  In your revision ensure you cite all your sources (including your own works), and quote or rephrase any duplicated text outside the Methods section. Further consideration is dependent on these concerns being addressed. | We have revised them accordingly. |
|  | Please organise your Abstract in subheadings. | We have added the subheadings. |
|  | We note that you have indicated that data from this study are available upon request. PLOS only allows data to be available upon request if there are legal or ethical restrictions on sharing data publicly. For information on unacceptable data access restrictions, please see <http://journals.plos.org/plosone/s/data-availability#loc-unacceptable-data-access-restrictions>. | We have provided the anonymized data set used for the analysis |
|  | In your revised cover letter, please address the following prompts:    a) If there are ethical or legal restrictions on sharing a de-identified data set, please explain them in detail (e.g., data contain potentially identifying or sensitive patient information) and who has imposed them (e.g., an ethics committee). Please also provide contact information for a data access committee, ethics committee, or other institutional body to which data requests may be sent.    b) If there are no restrictions, please upload the minimal anonymized data set necessary to replicate your study findings as either Supporting Information files or to a stable, public repository and provide us with the relevant URLs, DOIs, or accession numbers. Please see <http://www.bmj.com/content/340/bmj.c181.long> for guidelines on how to de-identify and prepare clinical data for publication. For a list of acceptable repositories, please see <http://journals.plos.org/plosone/s/data-availability#loc-recommended-repositories>.  We will update your Data Availability statement on your behalf to reflect the information you provide. | We have provided the anonymized data set used for the analysis |
|  | Please include captions for your Supporting Information files at the end of your manuscript, and update any in-text citations to match accordingly. Please see our Supporting Information guidelines for more information: http://journals.plos.org/plosone/s/supporting-information. | We have included the captions and in text citations accordingly. |
