# Supplementary material for: Quality of reporting of cranial irradiation techniques in randomized controlled trials of primary brain tumors: A systematic review
Source: PLoS One. 2020 Nov 5;15(11):e0241566. doi: 10.1371/journal.pone.0241566 (PMC7644083; doi:10.1371/journal.pone.0241566)
Supplement: Attachment — Submitted filename: Response to reviewers 2.docx [file pone.0241566.s008.docx]

Response to reviewers

| No. | Reviewer | Reviewer comments | Our response |
| --- | --- | --- | --- |
| 1 | 1 | Thanks for submit your revised version of manuscript about "Quality of reporting of cranial irradiation techniques in randomized controlled trials of primary brain tumors: a systematic review" In my opinion you did not write your article correctly and it should be rewrite principally. | Thank you for your feedback.  We have rewritten several sentences to describe each section of the manuscript more clearly as shown in the revised manuscript with the tracked changes.  We have also employed the Grammarly software to assist with the grammatical edits. |
| 2 | 2 | The authors have addressed all my comments, which helped to improve the manuscript. The methodology is more clearly described, which facilitates interpretation of the results. My only concern is the English language. I would advise to involve a native speaker for grammatical edits. | We have rewritten several sentences to describe each section of the manuscript more clearly as shown in the revised manuscript with the tracked changes.  We have also employed the Grammarly software to assist with the grammatical edits. |
